# Integrative genome-wide expression profiling identifies three distinct molecular subgroups of renal cell carcinoma with different patient outcome

**DOI:** 10.1186/1471-2407-12-310

**Published:** 2012-07-23

**Authors:** Manfred Beleut, Philip Zimmermann, Michael Baudis, Nicole Bruni, Peter Bühlmann, Oliver Laule, Van-Duc Luu, Wilhelm Gruissem, Peter Schraml, Holger Moch

**Affiliations:** 1 Institute of Surgical Pathology, University Hospital Zurich, Schmelzbergstrasse 12, 8091, Zurich, Switzerland; 2 Department of Biology, ETH Zurich, Universitätstrasse 2, 8092, Zurich, Switzerland; 3 Institute of Molecular Life Sciences, University of Zurich, Winterthurerstrasse 190, 8057, Zurich, Switzerland; 4 Seminar for Statistics, ETH Zurich, Rämistrasse 101, 8092, Zurich, Switzerland; 5 PAREQ Research AG, Wagistrasse 14, 8952, Schlieren, Switzerland

**Keywords:** DNA-microarray, SNP-array, RCC subgroups, Tissue microarray, Outcome

## Abstract

**Background:**

Renal cell carcinoma (RCC) is characterized by a number of diverse molecular aberrations that differ among individuals. Recent approaches to molecularly classify RCC were based on clinical, pathological as well as on single molecular parameters. As a consequence, gene expression patterns reflecting the sum of genetic aberrations in individual tumors may not have been recognized. In an attempt to uncover such molecular features in RCC, we used a novel, unbiased and integrative approach.

**Methods:**

We integrated gene expression data from 97 primary RCC of different pathologic parameters, 15 RCC metastases as well as 34 cancer cell lines for two-way nonsupervised hierarchical clustering using gene groups suggested by the PANTHER Classification System. We depicted the genomic landscape of the resulted tumor groups by means of Single Nuclear Polymorphism (SNP) technology. Finally, the achieved results were immunohistochemically analyzed using a tissue microarray (TMA) composed of 254 RCC.

**Results:**

We found robust, genome wide expression signatures, which split RCC into three distinct molecular subgroups. These groups remained stable even if randomly selected gene sets were clustered. Notably, the pattern obtained from RCC cell lines was clearly distinguishable from that of primary tumors. SNP array analysis demonstrated differing frequencies of chromosomal copy number alterations among RCC subgroups. TMA analysis with group-specific markers showed a prognostic significance of the different groups.

**Conclusion:**

We propose the existence of characteristic and histologically independent genome-wide expression outputs in RCC with potential biological and clinical relevance.

## Background

Renal cell carcinoma (RCC) represents the most common malignancy arising in the adult kidney, with increasing incidence and poor prognosis [1]. RCC can be pathologically subdivided into different histological subtypes [2] based on the microscopic phenotype and the presence or absence of von Hippel-Lindau (*VHL*) gene alterations. The most frequent histological subtype is clear cell RCC (ccRCC), followed by papillary RCC (pRCC) and chromophobe RCC (chRCC). Important prognostic parameters for RCC involve tumor and nodal stage [3].

In the search of critical genes, molecular studies identified several onco- and tumorsupressor gene candidates that are mutated and/or located within frequently gained and lost chromosomal regions of RCC [4-9]. Although multiple genes and signaling pathways have been implicated in renal cancer, *VHL* is the best characterized driver mutation, as it is mutated in the majority of sporadic ccRCC [10]. Loss of function of the VHL protein (pVHL) in ccRCC culminates in the deregulation of downstream target pathways that are important for uncontrolled cell proliferation and malignant progression [11]. In addition to *VHL,* alterations of the genes *MET*, *FH* and *BHD* are thought to be responsible for the development of familial RCC [2]. The low mutation frequencies reported for these genes in sporadic RCC subtypes [12-14], however, suggest other genes and pathways being relevant for the vast majority of RCC.

Microarray technology is an efficient approach to get comprehensive insights into individual and common tumor type-specific expression patterns based on hundreds of informative genes. Previous gene expression analyses using DNA microarrays suggested that unsupervised clustering combined with supervised learning methods optimize the molecular (re)classification of RCC to better predict cancer behavior. Distinct molecular expression profiles distinguishing between good and bad prognosis in RCC were identified [15-26]. However, the tumor samples were pre-selected according to histologic, clinical or molecular criteria in most of these studies. As a consequence, attempts to interpret general molecular strategies of RCC may have therefore been concealed by the co-appearance of surrogate markers. For example, although ccRCC is phenotypically and genotypically clearly different from pRCC and chRCC, we hypothesized that similar sets of common functional capabilities may exist in these tumor subtypes characterizable by the sum of molecular features occurring in RCC, irrespective of any histologic, clinical or single molecular parameters.

To test this hypothesis, we applied unsupervised clustering methods and integrated gene expression-, SNP and tissue microarray data using two independent sets of 146 and 254 RCC, respectively.

## Methods

### Renal cancer tissue, cell lines and nucleic acid extraction

Frozen primary RCC and tissue from RCC metastases were obtained from the tissue biobank of the University Hospital Zurich. This study was approved by the local commission of ethics (ref. number StV 38–2005). All tumors were reviewed by a pathologist specialized in uropathology (H.M.), graded according to the Fuhrman grading system and histologically classified according to the World Health Organization classification [2]. All tumor tissues were selected according to the histologically verified presence of at least 80% tumor cells. Total RNA was extracted from 74 ccRCC, 19 pRCC, 2 chRCC, 2 mixed cc/pRCC and 15 metastases of ccRCC using the RNeasy minikit (Qiagen, Hilden, Germany). The quality of the RNA was measured using the Agilent Bioanalyzer 2100. DNA was extracted from 56 ccRCC, 13 pRCC and 69 matched normal renal tissues using the Blood and Tissue Kit (Qiagen). Expression analysis was additionally performed with RNA from 24 RCC cell lines, 6 cell lines from RCC metastasis and 4 prostate cancer cell lines as controls. All tumors and cell lines considered in expression and SNP-array experiments are listed in the supplementary data (see Additional file 1: Table S1).

### Microarrays and expression analysis

Reverse transcription of RNA, DNA labeling and hybridization on HG-U133A High-Throuput Arrays (Affymetrix, Santa Clara, USA) were performed at the Broad Institute of MIT and Harvard Medical School (Cambridge, MA, USA). Arrays were scanned using the HT Scanner. Affymetrix GeneChip data was normalized using MAS5 from Bioconductor [27] and log_2_-scaled. Hierarchical clustering was done with TIGR MeV [28] using Euclidian distance and average linkage. The identification of tumor type-specific biomarkers was performed using SAM [29]. The most significant genes were cross-checked in GENEVESTIGATOR [30] to remove probe sets that had absent calls across all samples. GENEVESTIGATOR is an online platform based on a high quality, manually curated database of microarray experiments enabling gene expression and regulation studies as well as the search for groups of genes sharing similar expression patterns by means of clustering and biclustering algorithms.

Generation and analysis of gene sets were performed with the PANTHER (Protein Analysis Through Evolutionary Relationships; http://www.pantherdb.org) Classification System database [31], by considering both, PubMed & Celera, datasets. The global functional overview of 17,181 human genes was extracted from PANTHER by using its standard settings. According to the developers, PANTHER is classifying all genes by their function through consideration of published scientific evidence and/or evolutionary relationships, therefore being able to even predict a function also in the absence of experimental evidence.

**Figure 1:**
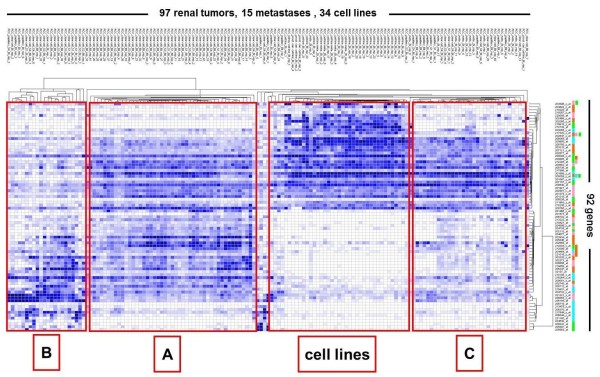
**Molecular subclassification of renal cell carcinoma.** Two-way hierarchical clustering of Affymetrix gene expression microarray data of 146 samples against the 92 pathway-related genes. Blue: relative increase-, white: relative decrease of gene expression. The PANTHER “pathway” affiliation of probe sets is indicated by colored barcode (right): green – “Inflammation”; pink – “Wnt”; orange – “Angiogenesis” and light blue – “Integrin” (see also Additional file 3: Table S2). Note: None of the 4 groups is exclusively related to any of the “dominating pathways”.

Affymetrix probe sets were identified for at least half of the genes extracted from PANTHER. For each gene set, a two-way hierarchical clustering of probe sets versus the complete set of expression arrays (146 arrays shown in Additional file 1: Table S1) was run using GENEVESTIGATOR. We selected up to four clusters that best represented the overall array clustering in each pathway (see Additional file 2: Figure S1 A-D). Finally, a joint clustering of all probe sets from these clusters resulted in the groupings described (Figure 1).

### Statistical data validation of the three tumor groups

Random Forest and linear discriminant analysis were calculated using packages *random Forest*[32] and MASS *lda*[33] of R version 2.11.1, respectively. The testing of significance of variable selection was performed by group label shuffling; meaning by calculating the probability of finding the same classification accuracy for random groups with the same number of variables. Therefore, group labels were shuffled randomly 500 times, each time random forest was calculated, the variables were sorted by relevance and the 4 best variables were then used for recalculation of random forest with same shuffled groups. Clustering was performed using function *hclust* R version 2.11.1.

### SNP array analysis and classification

Labeling and hybridization of extracted DNA on Genome Wide Human SNP 6.0 arrays (Affymetrix) were performed at the Broad Institute of MIT and Harvard Medical School (Cambridge, MA, USA). Arrays were scanned using the GeneChip Scanner 3000 7 G. Raw probe data CEL files were processed with the R statistical software framework using the array analysis packages from the aroma.affymetrix project [34]. Total copy number estimates were generated using the CRMAv2 method [35] including allelic cross talk calibration, normalization for probe sequence effects and normalization for PCR fragment-length effects. Copy number segmentation was performed using the Circular Binary Segmentation method [36] implemented in the DNA copy package available through the Bioconductor project. Normalized data plots including segmentation results, oncogene map positions and known copy number variations as reported in the Database of Genomic Variants were generated with software packages developed for the Progenetix project [37]. Map positions were referenced with respect to the UCSC genome assembly hg18, based on the March 2006 human reference sequence (NCBI Build 36.1). Data from arrays with prominent probe level noise after normalization were excluded before proceeding with the evaluation of copy number imbalances. Overall, 114 SNP 6.0 arrays (45 RCC and 69 normal tissue samples) were used for final data processing.

For the generation of overall genomic imbalance profiles, probabilistic thresholds of 0.13/-0.13 were used for genomic gains and losses, respectively. Microarray and SNP data have been deposited in GEO under GSE19949.

### Tissue microarray construction and immunohistochemistry

We used two TMAs with tumor tissue from 27 and 254 RCC specimens, respectively. The samples were retrieved from the archives of the Institute for Surgical Pathology; University Hospital Zurich (Zurich, Switzerland) between the years 1993 to 2003. In addition to tumor stage and Fuhrman grade, information about sarcomatoid differentiation was also available for all tumors. Areas with sarcomatoid differentiation were identified by one pathologist (H.M.) and defined as described [38].

TMAs were constructed as previously described [39]. To sufficiently address tumor heterogeneity, we used 3 punches per tumor for the construction of the TMA with 27 tumor samples. One biopsy cylinder per tumor was regarded as sufficient for constructing the TMA with 254 tumors. TMA sections (2.5 μm) on glass slides were subjected to immunohistochemical analysis according to the Ventana (Tucson, AZ, USA) automat protocols. CD34 (Serotec Ltd. - clone QBEND-10, dilution 1:800), MSH6 (BD Biosciences – clone 44, dilution 1:500) and DEK (BD Biosciences – clone 2, dilution 1:400) stainings were performed and analyzed under a Leitz Aristoplan microscope (Leica, Wetzlar, Germany). Tumors were considered MSH6 or DEK positive if more than 1% of tumor cells showed unequivocal nuclear expression. MVD was determined as previously described [40]. Contingency table analysis and Pearson’s chi-square tests were used to analyze the associations between protein expression patterns and clinical parameters. Overall survival rates were determined according to the Kaplan–Meier method and analyzed for statistical differences using a log rank test. A Cox proportional hazard analysis was used to test for independent prognostic information. The statistics were performed with SPSS 18.0 for Windows (SPSS Inc., Chicago; IL).

## Results

### Gene expression patterns split RCC into three molecular groups

We chose PANTHER [31] to extract a standard overview of the classification of 17,181 human genes by their function. PANTHER allocates 6,017 of these genes into 145 “superior pathways”. Four of these pathways involve more than 150 genes (“Wnt” 497 genes, “Inflammation” 476 genes, “Angiogenesis” 354 genes, “Integrin” 365 genes), others such as “Cysteine biosynthesis”, listed only one gene.

We used the RNA extracted from 97 primary RCCs of different pathologic parameters, 15 RCC metastases and 34 cell lines (see Additional file 1: Table S1), to identify any gene expression patterns in pathways containing more than 20 genes. For this purpose, we used the gene expression data obtained from Affymetrix HG-U133A arrays and performed two-way hierarchical clustering with each of those gene sets using GENEVESTIGATOR [30].

Only within the matrices of “Wnt”, “Inflammation”, “Angiogenesis”, and “Integrin“, which included process-related as well as downstream target genes as suggested by PANTHER, we observed clearly distinguishable major gene expression clusters. The most prominent gene expression clusters are highlighted in Additional file 2: Figure S1 A-D and Additional file 3: Table S2. Interestingly, no such differentiating gene expression patterns were obtained through hierarchical clustering of the genes of the remaining pathways (i.e. apoptosis or HIF-signaling) which, according to PANTHER, contained less than 150 genes (see Additional file 2: Figure S1 E).

We next asked for possible relations among the different tumor groups and their specific gene expression patterns as detected from the 4 “dominating pathways”. For this purpose, we selected with GENEVESTIGATOR up to four gene clusters from each of the four matrices encompassing a total of 92 genes, which were most representative for the overall clustering of the samples within each matrix (see Additional file 2: Figure S1 A-D and Additional file 3: Table S2) and combined them into a new matrix. Subsequent clustering of this matrix yielded four distinct groups (Figure 1). Notably, the 92 genes represented only a small percentage of genes involved in the suggested “dominating pathways” (see Additional file 3: Table S2). Moreover, many of them (such as *MAPK*, *RHO*, *NOTCH*, *PDGF*, *RAS*, *JUN*, *ARF*, *PIK3*) also belong to other cancer-related pathways. Importantly, as none of the four groups was associated with any of those pathways (Figure 1 – color coding bar, right), we preferred to subdivide the groups into tumor groups “A”, “B”, “C” and “cell lines”. Table 1 shows the 97 RCC specimens subdivided in groups A, B and C and characterized by tumor subtype, tumor stage and nuclear differentiation grade. Most interestingly, primary RCC split into group A, B or C, irrespective of their clinical characteristics (see also Additional file 1: Table S1).

**Table 1:** Classification of two RCC sets and their clinical characteristics

		**RCC microarray set**				**RCC TMA set**			
		A	B	C		A	B	C	
		N (%)	N (%)	N (%)	N (total)	N (%)	N (%)	N (%)	N (total)
Histological	ccRCC	48 (65)	16 (22)	10 (13)	74	39 (27)	66 (45)	41 (28)	146
subtype	pRCC	1 (5)	6 (32)	12 (63)	19	0	17 (52)	16(48)	33
	chRCC	0	1 (50)	1 (50)	2	0	7 (70)	3 (30)	10
	cc/pRCC	0	0	2 (100)	2	-	-	-	-
Tumor stage	pT1/pT2	32 (52)	16 (26)	14 (22)	62	27 (28)	48 (51)	20 (21)	95
	pT3/pT4	17 (49)	7 (20)	11 (31)	35	12 (14)	39 (45)	36 (41)	87
Fuhrman grade	grade 1	3 (43)	1 (14)	3 (43)	7	1 (100)	0	0	1
	grade 2	27 (63)	8 (19)	8 (19)	43	20 (36)	20 (36)	15 (27)	55
	grade 3	18 (44)	12 (29)	11 (27)	41	16 (20)	44 (55)	20 (25)	80
	grade 4	1 (17)	2 (33)	3 (50)	6	2 (4)	24 (47)	25 (49)	51
sarcomatoid	yes	nd	nd	nd		3 (7)	20 (43)	23 (50)	46
	no	nd	nd	nd		36 (26)	67 (48)	37 (26)	140

nd: not done due to limited tissue material.

### Gene delineation for stable RCC stratification

**Figure 2:**
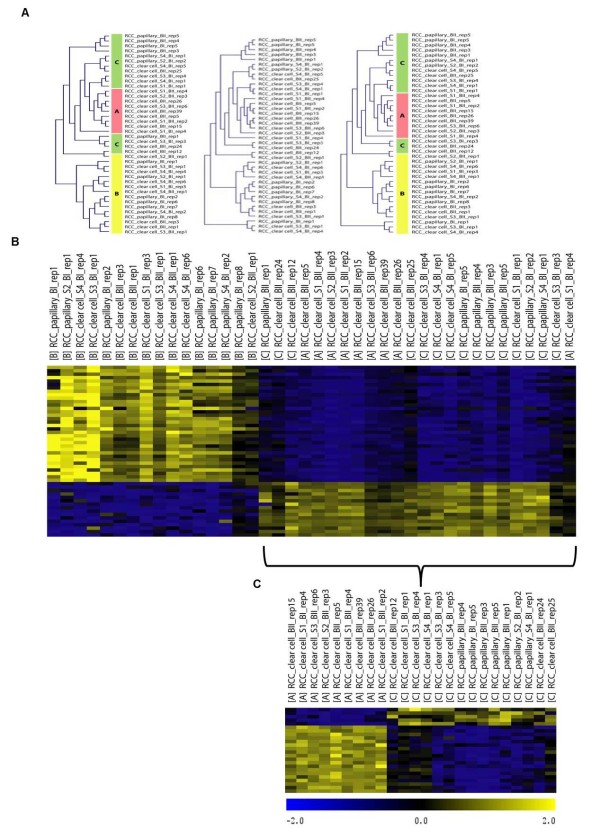
**Genome-wide expression signatures in RCC. A.** Hierarchical clustering of 40 RCC samples across all probe sets of the HG-U133A array, identifying the 3 groups (left). Hierarchical clustering of the 40 RCC samples based on expression signal values from 769 genes identified from the SNP array analysis, show diffuse clusters prior to group acquaintance (middle), but are unraveling the 3 RCC groups when individual tumors are affiliated (here: color coded) to their respective group before clustering (right). **B – C.** Heatmaps of RCC group-specific signatures with corresponding intensity bars (absolute values). Relative increase (yellow) and relative decrease (blue) of gene expression. **B**. Gene expression of the 50 best classifiers of subgroup B against subgroups A/C across a subset of A, B and C RCC. **C**. Gene expression of the 24 best classifiers of subgroup A against subgroup C across a subset of A and C RCC subgroups.

To confirm that the expression status of our 4 groups is specific, we profiled gene expression across 40 primary RCC samples arbitrarily chosen from the three RCC groups. Five independent hierarchical clusterings of these samples across arbitrarily chosen and pathway independent probe sets as well as a clustering against all 22,000 probe sets of the Affymetrix array showed that group B was clearly distinct from A and C. Notably, group A always appeared as a tight cluster within the C clad (Figure 2A left and Additional file 4: Figure S2). These findings confirmed the previous subgrouping of RCC based on the selected 92 genes (Figure 1) and moreover suggests the presence of genome wide, discrete and group-specific gene expression signatures.

### Identification of the best RCC group identifiers

By using SAM [29], at least a 2-fold change in the expression level was seen for more than 2,000 genes, with 1,455 genes being higher and 715 genes being lower expressed in group B compared to A/C, and 221 genes positively and 11 genes negatively regulated in A versus C.

The most differentially regulated genes between group B and groups A/C were represented by 48 genes, with 16 low expressed in B but strongly expressed in A/C (8.7 – 5.7 fold change) and 32 transcripts abundant in B but decreased in A/C (14.4 – 5.2 fold change) (Figure 2B; Additional file 5: Table S3). Twenty-three genes clearly distinguished groups A and C with 4 genes highly expressed in C but not in A (14.3 – 2.5 fold change), while 19 were highly expressed in A but not in C (16.0 – 4.2 fold change) (Figure 2C; Additional file 6: Table S4).

### Statistical significance of the three RCC groups

**Figure 3:**
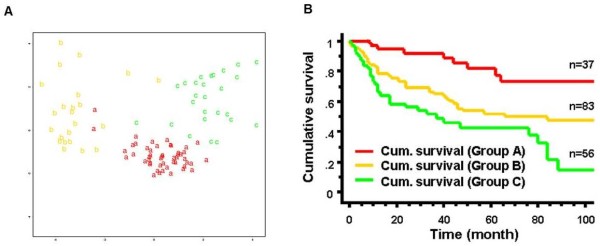
**Validation and prognostic significance of the genome-wide expression signatures in RCC. A.** Linear discriminant analysis of groups “A”, “B” and “C” with 4 selected variables (genes). Classification of the three groups using the 4 highest ranked variables of Random Forest allows linear discriminant analysis (LDA) with 96.94% accuracy. **B.** Kaplan–Meier analysis of tumor-specific survival in 176 RCC patients. Subgroup A (high MVD, DEK and MSH positive), B (high or low MVD, MSH6 negative) and C (low MVD, DEK and MSH positive) (log rank test: p < 0.0001).

The groups “A”, “B” and “C” were further investigated towards accuracy and reproducibility of their classification. Using Random Forest, classification accuracy reached 96.94% if only four variables out of the >22,000 measured genes were selected. The three groups separated very well as only 3 of the 97 measurements were misclassified under these conditions (Figure 3A). To test whether these three groups are outstanding, label shuffling of the groups and retrying classification with the four best variables was performed. Shuffled groups were analyzed for how often the same or a better classification accuracy than the original was achieved. Label shuffling considered that clusters A, B and C each contained 49 individuals and most “subtypes” in cluster A were ccRCC. Therefore at least one third of A was occupied with randomly selected ccRCC from B and C. 500 times label shuffling and classification trials resulted in zero times same or better classification accuracy (p < 0.002).

### Integration of DNA copy number alterations (CNAs) to the three RCC subgroups

In a first step, we analyzed genomic profiles of 45 RCC and corresponding normal tissues using Affymetrix 6.0 SNP arrays. We extracted an overall summary of detected genomic imbalances using Progenetix [37] and compared them to the entire available dataset of 568 RCC in the Progenetix database at censoring time (see Additional file 7: Figure S3A). Consistent with previous CGH data [41], our results confirmed the overall composite of CGH profiles in RCC.

In order to clarify whether our three RCC groups are characterized by combinations and/or frequencies of specific CNAs, we analyzed our groups using CNA data from 36 RCC for which high quality SNP- and gene expression microarray data were available and allocated and color coded with regard to Figure 1 (see also Additional file 1: Table S1), 20 tumors in group A, 3 in group B and 13 in group C (see Additional file 8: Table S5). By displaying all CNAs mapped to 811 cytogenetic bands (UCSC - hg18 cytogenetic mapping; chromosomes 1–22), all chromosomes were affected including the known RCC subtype-specific genomic alterations 3p-, 5q+(ccRCC) and 7+, 17+, 20+ (pRCC). This result is in line with previous CGH data [41]. Notably, loss of 3p was observed in all 3 groups and increased genomic derangements were seen in groups B and C compared to group A (see Additional file 7: Figure S3B).

Next, in order to identify the genes residing in minimally affected CNAs that possibly also directly contribute to the group-specific output signatures, we focused on tumor-specific genomic changes below 5 Mb which is the approximate resolution limit for chromosomal losses and gains obtained by chromosomal CGH [42]. We found 126 different regions in our cohort varying between 0.5 kb to 5 Mb and encompassing 61 allelic gains and 65 allelic losses (see Additional file 9: Table S6). These chromosomal regions harbored coding regions of a total of 769 genes. Interestingly, in contrast to large chromosomal aberrations commonly detected by CGH in public data sets, the genomic alterations <5 Mb could not be linked to morphologically defined RCC subtypes. By looking at all chromosomal changes occurring in our RCC set, we found a unique cytogenetic “fingerprint” characteristic for each tumor. Despite this uniqueness we were able to allocate all RCC to one of the 3 groups at the gene expression level (Figure 1).

Unsupervised hierarchical clustering of the 769 CNA-affected genes (see Additional file 9: Table S6) against the 40 arbitrarily selected primary RCCs (see chapter “Gene delineation”) showed rather diffuse RCC clusters (Figure 2A middle) indicating at first no direct linkage to the three RCC signatures. However, as it was already demonstrated with randomly, CNA non-affected, picked gene sets (see Additional file 4: Figure S2), the 769 CNA-affected genes could eventually be assigned to the three RCC groups, but only by knowing the three specific groups before clustering (Figure 2A right).

### Molecular RCC grouping is an independent, survival-associated prognostic factor

We finally asked whether RCC of the three groups could also be classified by characteristic morphologies or specific expression patterns on the protein level. For this purpose, we randomly selected 9 RCC from each of the three respective groups (Figure 1) and placed them into a small tissue microarray (TMA). A Hematoxylin/Eosin stained TMA section was blindly evaluated by a pathologist (H.M.). All nine tumors of group A were characterized by high microvessel density (MVD), whereas there were no specific morphologic features in the tumors of groups B and C. To further verify this finding, we immunohistochemically stained the endothelial cell marker CD34 in the 27 RCC. As shown in Additional file 10: Table S7, the results largely confirmed group-specific angiogenic traits. All nine tumors in group A, but only three in group B and one in group C had more than 100 microvessels, whereas the remaining ones had less than 50 microvessels per arrayed spot (0.036 mm^2^). Tumors with high and low MVD were classified accordingly. In order to find more group-specific markers which separate group B tumors from A/C and group A tumors from C in combination with the CD34 staining, we further searched genome wide with SAM [29] for genes with a clear present or absent expression profile in the three groups. SAM identified several candidates, including *DEK* and *MSH6*, for which well-established antibodies were available. By examining immunostaining patterns of several protein candidates coded by these genes, we were able to assign tumors with high MVD as well as DEK and MSH6 positivity to group A, high or low MVD and MSH6 negative tumors to group B, and tumors with low MVD but DEK and MSH6 positivity to group C. Examples of immunostained RCC are shown in Additional file 11: Figure S4.

To evaluate the obtained group-specific protein expression patterns in a much higher number of tumors, we screened a TMA with 254 RCC. By strictly applying the staining combinations obtained from the small test TMA, 189 tumors (75%) were clearly assigned to a specific group. The pathologic characteristics of the tumors assigned to the three RCC groups are shown in Table 1. There were organ-confined and metastatic RCC of different tumor subtype and nuclear differentiation grade with varying frequencies in these groups. To determine the clinical aggressiveness of these groups, we focused our analysis on 176 of 189 RCC samples on the TMA for which survival data were available. Kaplan-Meier analysis showed a highly significant correlation (log rank test: p < 0.0001) of group affiliation with overall survival, in which patient outcome was best in group A and worst in group C (Figure 3B). This result was independent from tumor stage and grade in a multivariate analysis (see Additional file 12: Table S8). By performing this survival analysis, we demonstrate that the molecular re-classification of RCC allows the identification of early stage tumors (pT1 and pT2) with high metastasizing potential associated with poor patient prognosis. In addition, the finding of late stage RCC in group A also suggests the existence of patients with a relative good prognosis although their tumors were categorized as pT3.

## Discussion

In this study we used unsupervised hierarchical clustering and gene expression pattern combination approaches to detect robust molecular clusters which classify RCC into three molecular groups with distinct prognostic values. In many previous studies, RCC cases were either preselected or expression data were linked according to pathologic and clinical criteria for further analysis [15-26]. Potential markers may have therefore represented surrogate traits, overall confirming phenotypes at a molecular basis. To our knowledge, patterns of gene expressions, independent of pathological or single molecular parameters pointing to general RCC biology remained uncovered to date.

### Classification system databases suitable for comprehensive gene expression clustering

To identify common RCC gene expression signatures, we searched for large gene sets using the classification systems INGENUITY ( http://www.ingenuity.com/), KEGG ( http://www.genome.jp/kegg/) and PANTHER ( http://www.pantherdb.org). Our gene expression analyses demonstrated that more than 150 genes are required to obtain major and clearly distinguishable gene clusters. In contrast to Ingenuity and KEGG, only PANTHER is able to integrate several hundred genes into “superior pathways”. Only within the clustering of these four dominating processes different major group patterns were obtained. The number of genes in the remaining pathways was too low and therefore not suitable for cluster analysis. It is important to understand that it was not our intention to analyze specific pathways within RCC. We rather used this platform to visualize sets of gene expression clusters which are differentially regulated within different RCC. There was no notable association between any of the RCC groups and any of the 4 pathways. The 92 genes extracted from the 4 matrices (pathways) were rather equally distributed over the 3 RCC groups suggesting the partial involvement of all 4 pathways in the RCC groups. In our opinion the results of the clustering by using randomly selected 5 sets of about 700 genes clearly indicate that we could have taken any arbitrary chosen gene list for clustering independent of any pathways.

### Unsupervised versus supervised clustering

For supervised analysis of gene expression patterns in tumors algorithms are commonly used that are linked to known clinical parameters such as tumor subtype, metastatic-nonmetastatic or treated-untreated. Consequently, the number of clusters to be expected is already known. As we tried to identify non-biased gene expression patterns, we chose unsupervised analysis for which the resulting numbers of clusters are unknown. To circumvent this problem we combined the strongest gene expression patterns into a new matrix and re-clustered them by using the second clustering step, importantly, against the same tumor cohort ( Additional file 2: Figure S1 A-D and Figure 1). Our approach to randomly select genes and re-cluster them demonstrated that the three tumor groups remained stable ( Additional file 4: Figure S2). We therefore believe that our two-times-two-way non-supervised clustering method is an alternative strategy to re-classify tumor types independent of TNM criteria. We cannot rule out that additional groups exist which may appear if more samples are included in the analysis.

### Molecular signatures strictly separate RCC tissue from RCC cell lines

Surprisingly, the expression signature yielded from the renal cancer cell lines was clearly distinguishable from those derived from renal cancer tissues. We observed that individual cell line expression profiles, independent of their respective primary tumors, were all similar to each other. This general finding may mainly be caused by culture conditions, the artificial environment and the two-dimensional structure of cell culture layers. We therefore believe that expression profiling using cell lines would never lead to the detection of common renal cancer tissue-specific signatures. This also raises concerns about the possibility of discovering novel strategies for diagnosis and therapies by using *in vitro* systems only.

### Molecular signatures do not coincide with pathologic criteria

In contrast to the cell lines which represent a separate group, RCC metastases and primary RCC split into group A, B or C, irrespective of the tumor subtype, stage, differentiation grade or sarcomatoid differentiation. When looking at RCC group A, which contains almost only ccRCC, it seems that the clustering results correlate with the histological subtype. However, these ccRCC were of different tumor stage and grade. The same is true for the tumors in group B and C. In these groups ccRCC, pRCC as well as chRCC of different pathologic parameters were allocated. Furthermore, our molecular classification allows to additionally refine the staging and grading of tumors. Organ-confined RCC, particularly pT1 tumors, generally considered to have a good prognosis can further be subdivided in group A (good), B (worse) or C (worst) which also may have predictive impacts. Although ccRCC, pRCC and chRCC have a different morphological background, the combined appearance of the three histological subtypes across different clusters suggests molecular and functional similarities.

### The three RCC output signatures are not influenced by the VHL/HIF axis

Based on the results obtained from a series of previous *VHL* mutation analyses, it is widely accepted that the loss of function of pVHL mainly contributes to the development of ccRCC [43]. The inactivation of pVHL leads to HIF-α stabilization and, hence, to the upregulation of a number of genes involved in RCC progression (i.e. *VEGFA, PDGF, TGF, CXCR4, CA9*) [44-46]. Therefore, we assumed to detect gene expression patterns connected to HIF signaling pathways. However, gene expression patterns demonstrated no remarkable linkage between HIF-regulated pathways and any of the RCC subgroups. This finding is in line with the results of a recent study in which *VHL* wild-type tumors, HIF-1α and HIF-2α overexpressing tumors, as well as HIF-2α-only overexpressing tumors were found in both ccRCC clusters [26].

We also looked at the *VHL* mutation status in all analyzed ccRCC and identified gene sequence alterations in the majority of the tumors [10]. A recent study demonstrated that the thermodynamic stability and the functionality of pVHL is dependent on the location and the type of mutation [10]. As the frequencies and types of *VHL* mutations were similar in all three RCC groups, it was not surprising that there was no association with the gene expression patterns, neither with the *VHL* mutation status nor with any HIF-driven pathways (data not shown). Our data strongly suggest the existence of pVHL-independent mechanisms, resulting in distinct gene expression outputs which reflect common biologic pathways in renal cell cancer.

### RCC gene expression signatures are not directly linked to copy number alterations

Our integrative approach that combined SNP- and microarray data, revealed no direct correlation between the signatures of CNA-affected genes analyzed in 45 RCC and the three RCC groups. Only one of the 92 cluster forming genes (*ITGAL*; see Additional file 3: Table S2) belonged to the 769 genes residing within the 126 CNAs found in our RCC set. Moreover, hierarchical clustering of both CNA-affected and non-affected genes demonstrated that the three RCC gene expression patterns are not directly influenced by copy number alterations. This finding is in line with a recent study which also found many discrepancies between CNA and gene expression [47]. The authors suggest that the expression of many “driver” genes are less correlated with their copy number than “passenger” genes due to selective pressure. Additional multiple ways exist to up- or down-regulate a gene.

### RCC is not caused by alteration of single genes and pathways

It is remarkable that, although type and frequencies of CNAs were largely differing within the tumor cohort and varied between none (!) and 18 altered genomic regions in single tumors, each of the three group-specific gene expression patterns remained stable. We postulate that each of these RCC must have developed individual mechanisms in addition to CNAs (i.e. mutations, methylations, transcriptional and translational modifications), which together support the regulation of molecular components to reach one of the three tumor groups. A recent study showing that low CNA rates in tumors are related to increased levels of global DNA methylation and *vice versa*[48] supports our hypothesis.

In contrast to previous approaches, we combined several subtypes of RCC for non-supervised hierarchical clustering approaches in combination with LDA entirely unbiased from different clinico-pathologic parameters. Our results demonstrate that RCC group formation patterns remained very similar across various sets of genes arguing for a substantial number of genes which participate in the molecular definition of a RCC group. It is therefore not surprising that more than one third of the human genes have already been identified as being cancer-relevant [49] and many of them being claimed as potential biomarkers [50]. As a consequence, we believe that in a tumor many molecular pathways must be directly or indirectly affected to eventually reach one of the three output signatures.

### Characterization of the three RCC groups at the protein level

By subsequently performing our TMA analysis on a second, larger cohort of RCC we validated our results also on the protein level. To find appropriate markers we tested several antibodies directed against proteins whose genes were clearly upregulated in one of the groups. Among 10 candidates tested only MSH6, a DNA mismatch repair enzyme, and DEK, a chromatin- and RNA-associated protein mutated or overexpressed in certain cancers, showed reliable immunostaining results. The third protein, CD34, was indirectly identified by retrospectively analyzing the tumors histologically *after* the clustering analyses (Figure 1). We found increased microvessel density in group A by selecting the RCC samples randomly without knowing any specific pathological features (with the exception of stage and grade). Although not expressed in RCC cells, this endothelial marker is an ideal marker to morphologically distinguish group A from group B and C. Our effort to select suitable protein markers for the RCC groups demonstrated strong differences between the expression signatures at the RNA and the protein levels. Further protein analyses are needed to identify additional markers or marker combinations with both prognostic and predictive value.

## Conclusion

We believe that the identified genome-wide signatures point to common molecular programs characteristic for the biology of RCC. Here, we provide a novel concept for RCC classification implying potential impacts on tumor diagnostics and the development of tailor-made therapies. We still do not know whether the identified signatures are restricted to RCC or exist also in other cancer types. If the latter is true these expression patterns may represent outputs of molecular events which have led to common functional characteristics of cancers.

## Competing interests

The authors declare that they have no competing interests.

## Authors’ contributions

MBe and PS designed the study, analyzed, combined and interpreted data, and drafted the manuscript. PZ, OL and WG provided technical support, processed and analyzed expression array data. MBa provided technical support, processed raw SNP data and prepared Gene Expression Omnibus (GEO) information. NB and PB performed statistical significance analyses. V.D.L. prepared expression array analyses and extracted nucleic acids. HM and WG designed the study and HM reviewed all tumors. All authors read and approved the final manuscript.

## Pre-publication history

The pre-publication history for this paper can be accessed here:

http://www.biomedcentral.com/1471-2407/12/310/prepub

## Supplementary Material

Additional file 1 Table S1 List of samples used in expression array, 55 of them were also used for SNP experiment. Click here for file

Additional file 2 Figure S1 The strategy to find group-specific expression signatures in RCC. Hierarchical clustering of HG-U133A microarray probe sets representing genes from the Angiogenesis **(A)**, Inflammation **(B)**, Integrin **(C)**, and Wnt **(D)** “pathways” as annotated by PANTHER, across a set of 146 microarrays from our RCC experiment. For each “pathway”, up to four probe set clusters (red boxes) were selected and combined for subsequent re-clustering. **(E)** Another PANTHER “pathway” (Apoptosis) and one RCC-relevant “pathway” (HIF). Note the presence of less genes in these matrices compared to A-D and the absence of clear probe set clusters (except for cell lines in “Apoptosis”, indicated by the green bottom line), visually subdividing the matrix. Click here for file

Additional file 3 Table S2 List of clusters and containing genes, picked from separate "pathway clusterings" to be combined into one matrix. Click here for file

Additional file 4 Figure S2 The three RCC gene expression signatures spread genome-wide. Hierarchical clustering of 5 times arbitrarily chosen probe sets (each composed of ca. 660 genes) against group affiliated tumors (individual group-sample is labeled as A_, B_ or C_) **(A-E).** Note the tumor-group forming coincidence within the 5 independent analyses and the similarity with that shown in Figure 2A. Click here for file

Additional file 5 Table S3 List of top 48 genes with expression values, specific for RCC tumors of group B, relative to A and C. Click here for file

Additional file 6 Table S4 List of top 23 genes with expression value, distinguishing RCC tumors of group A from group C. Click here for file

Additional file 7 Figure S3 The landscape of CNAs in RCC does not correlate with novel molecular subgroups. **(A**) Regional genomic CNAs in RCC shown as percentage of analyzed cases (genomic gains: yellow, up; losses: blue, down). Top: depiction of the overall CNAs in the 45 study cases; Down: published chromosomal and array CGH RCC data accessible through the Progenetix database (568 cases). Copy number variants (CNVs) were not filtered from the study case data besides application of a 100 kb size limit. Note the similar profiles. **(B)** Case specific regional copy number imbalances in 36 RCC study cases with regional genomic gain or loss status matched to 811 cytogenetic regions. The genomic profiles are randomly arranged within their subtypes. White areas indicate concurrent gain and loss in this cytoband. Note the appearance of known subtype-specific genomic alterations (3p deletions, 5q gains identifying clear cell RCC – asterisk and arrow/left side; gains of chromosomes 7, 17 and 20 identifying papillary RCC - arrows right side). Click here for file

Additional file 8 Table S5 List of 36 RCC tumors considered on expression- and SNP array, and their affiliation to a specific group according to gene expression array. Click here for file

Additional file 9 Table S6 List of tumor-specific regions (0–5 Mb) and involved genes, identified by SNP experiment. Click here for file

Additional file 10 Table S7 The Test Tissue Microarray to establish antibody combinations for tumor/group affiliations. Click here for file

Additional file 11 Figure S4 Examples of immunostained RCC group-specific markers CD34, DEK and MSH6. ccRCC with CD34-stained vascular microvessels (A, B); ccRCC with strong nuclear DEK (C) and MSH6 (D) positivity. Click here for file

Additional file 12 Table S8 Cox proportional hazard regression analysis for survival. Click here for file
