# Supplementary figures and images for: Integrative genome-wide expression profiling identifies three distinct molecular subgroups of renal cell carcinoma with different patient outcome
Source: BMC Cancer. 2012 Jul 23;12:310. doi: 10.1186/1471-2407-12-310 (PMC3488567; doi:10.1186/1471-2407-12-310)

**Additional file - Fig. S1, A-E**

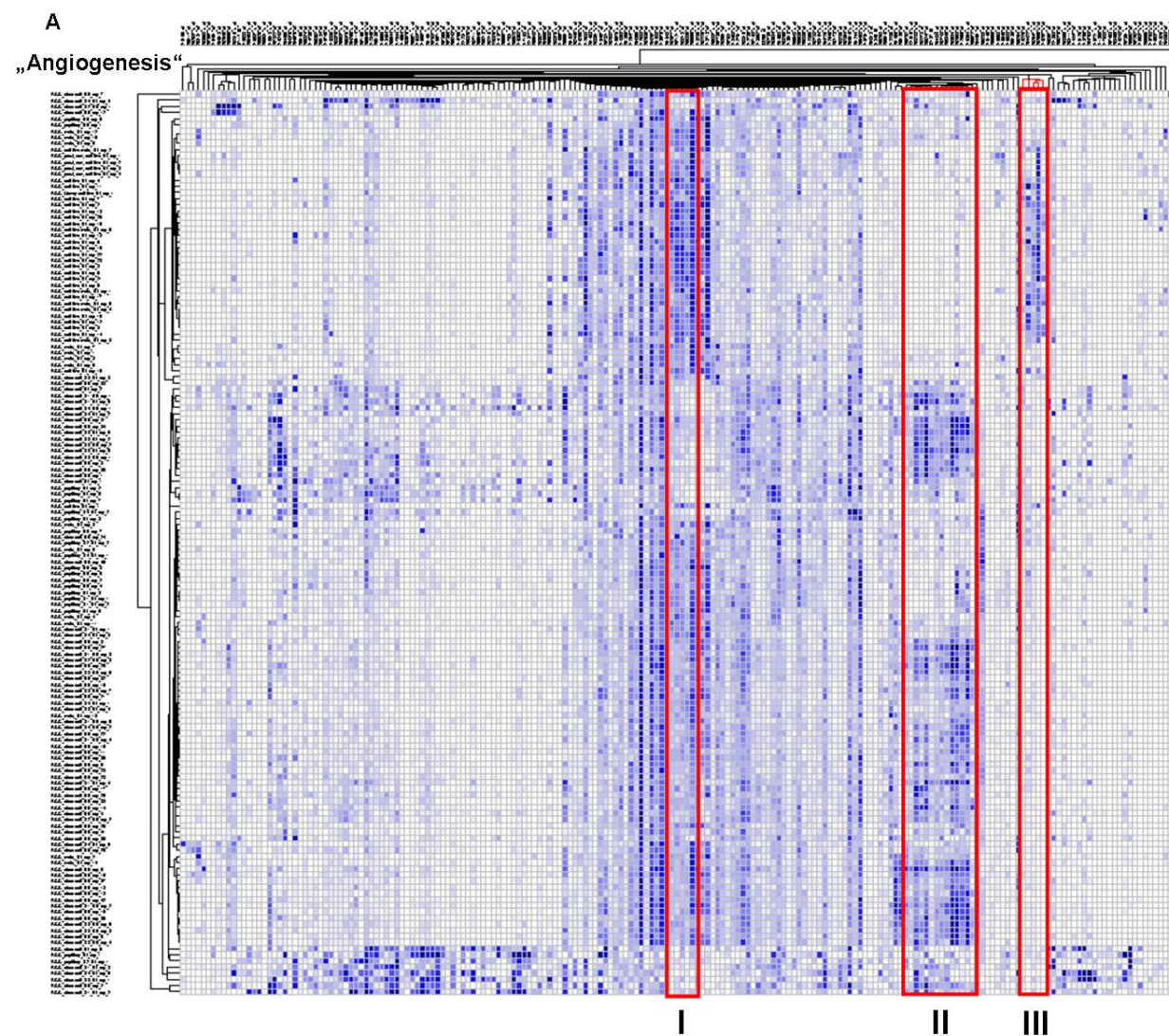

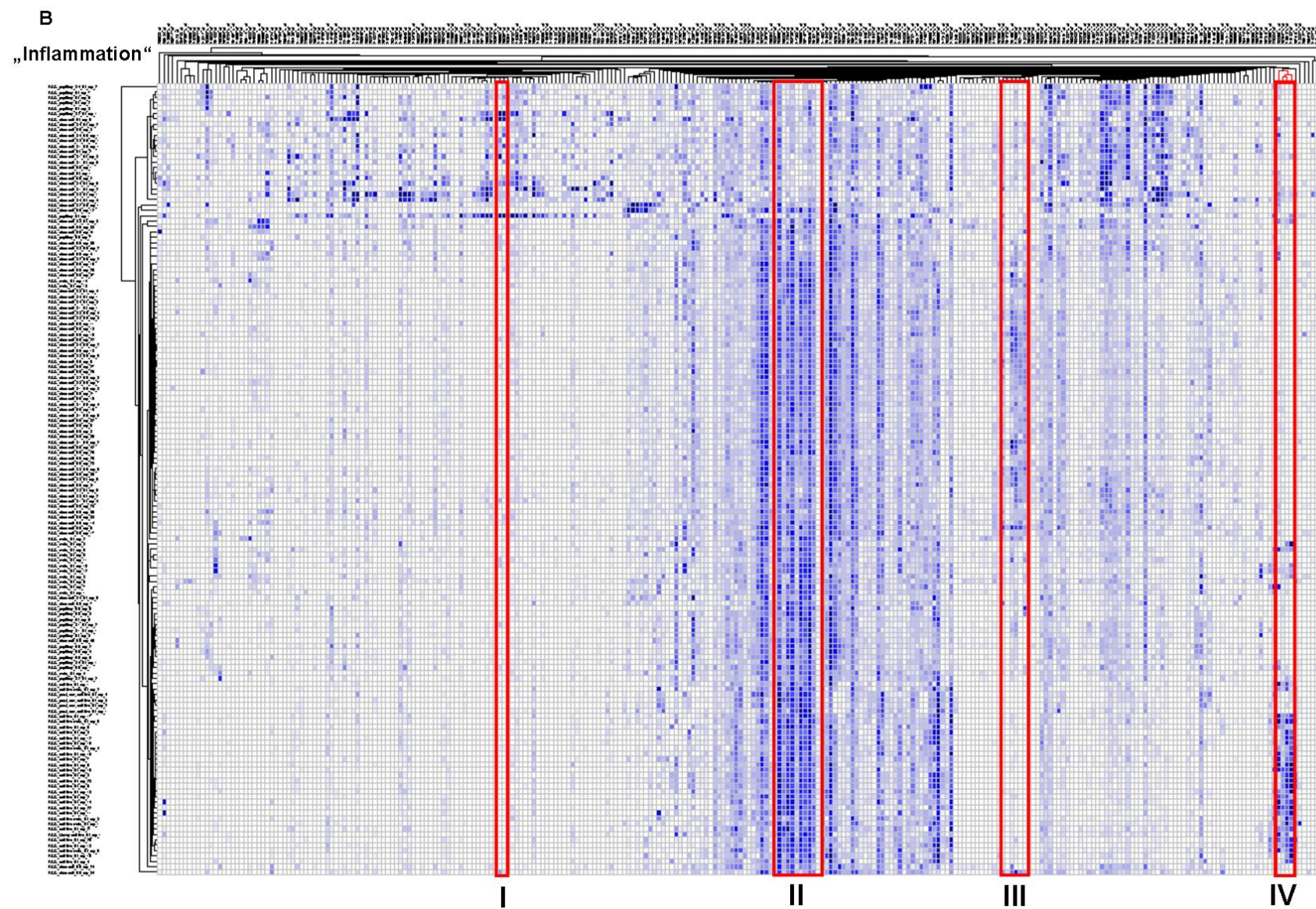

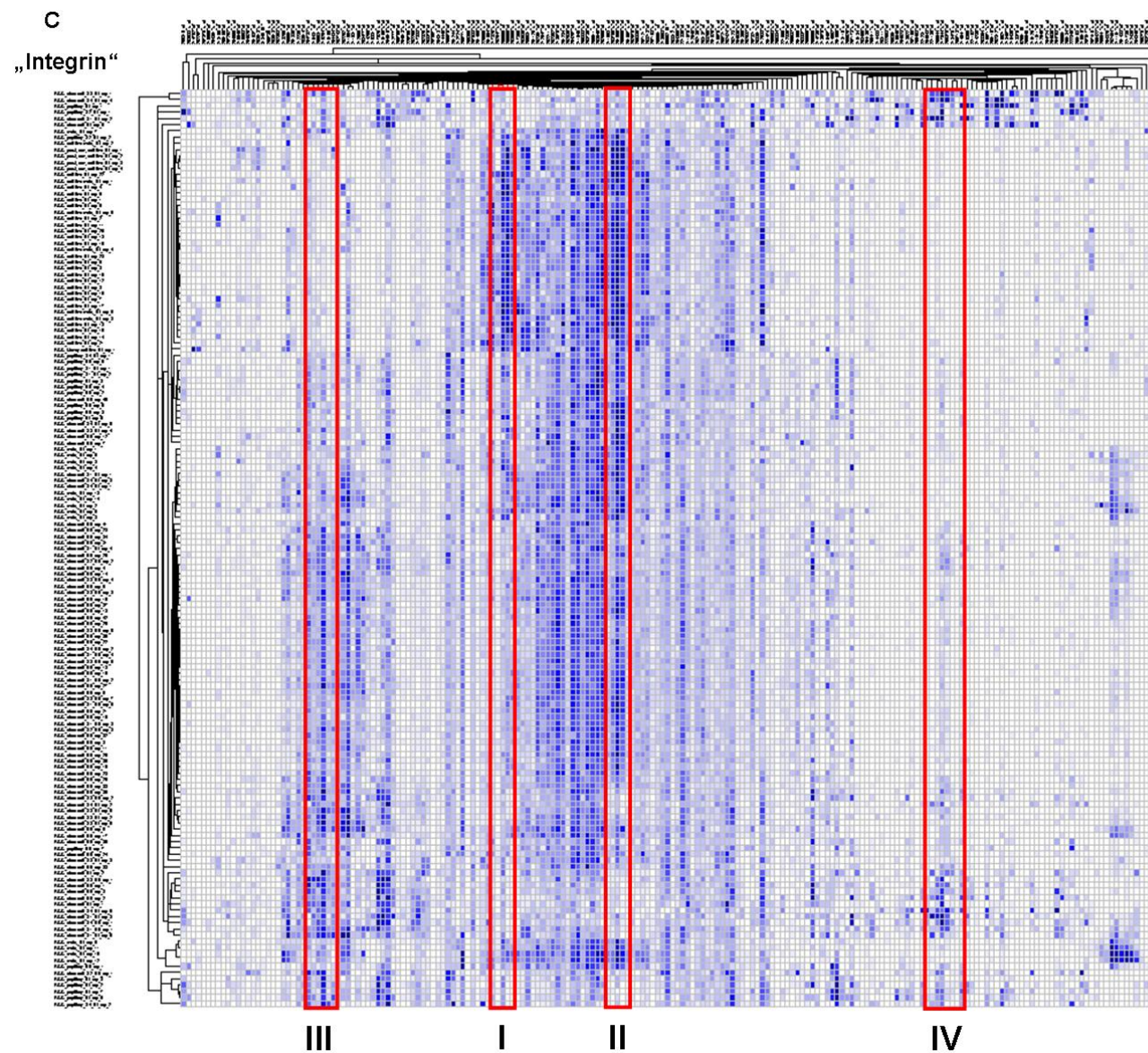

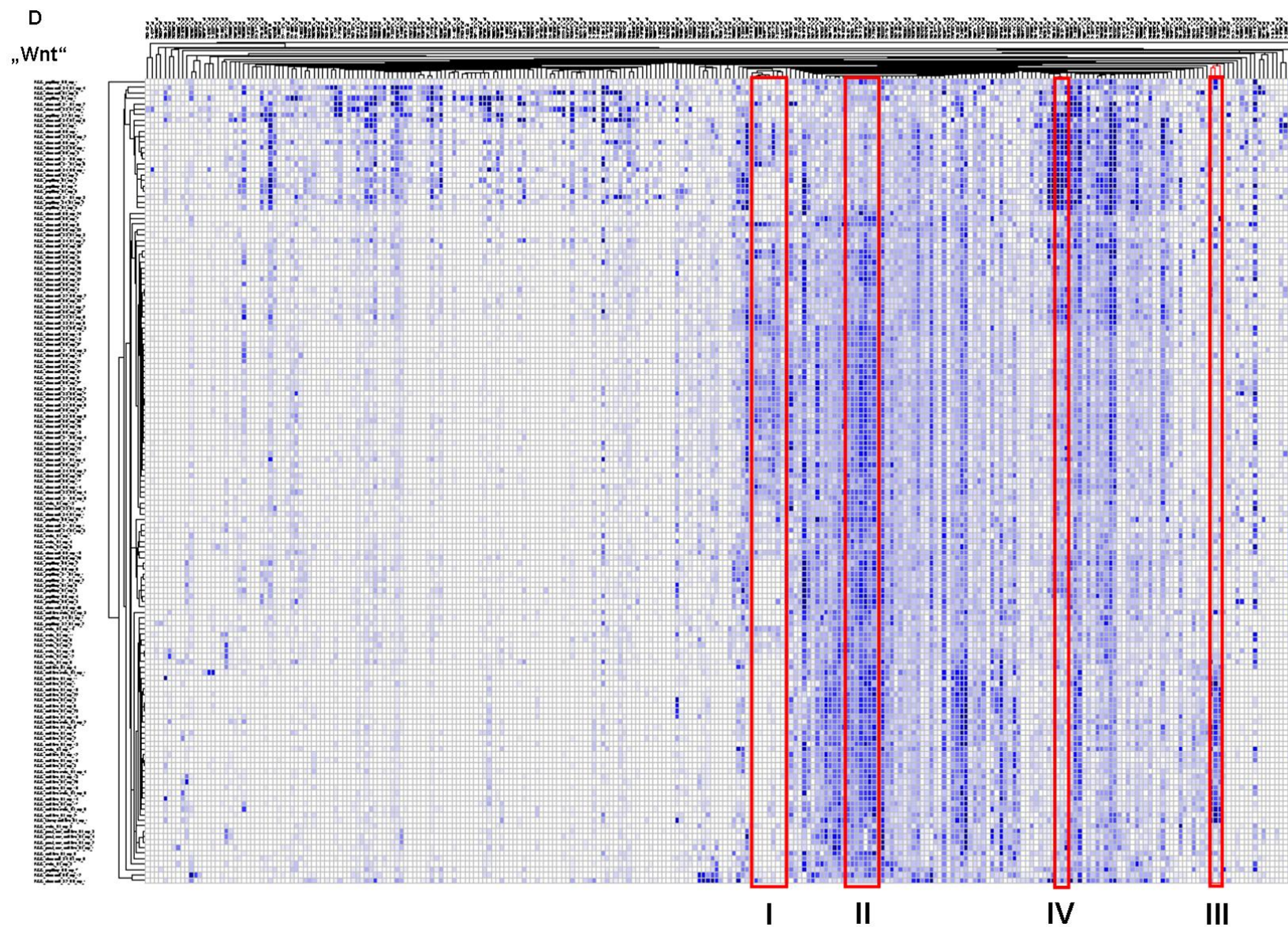

E

„Apoptosis“

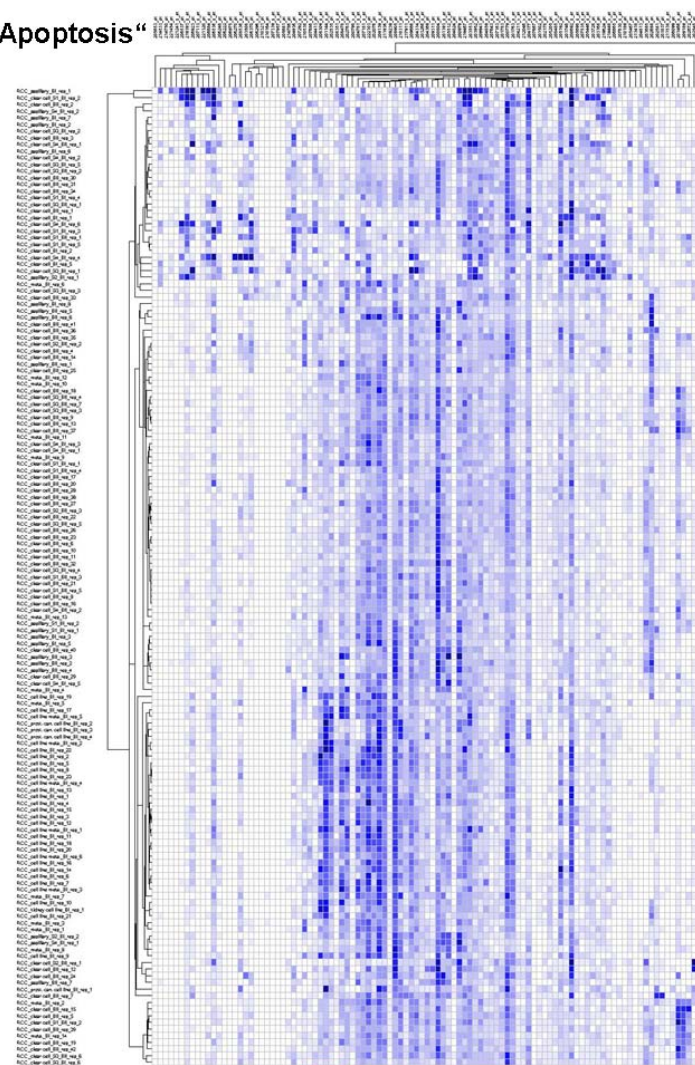

„HIF“

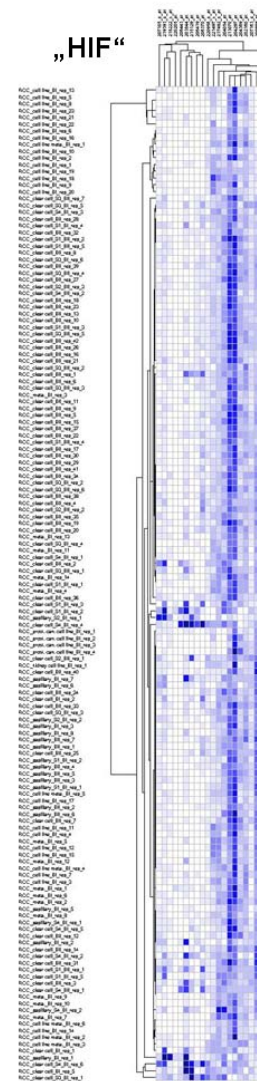

Supplement: Additional file 2 Figure S1 — The strategy to find group-specific expression signatures in RCC. Hierarchical clustering of HG-U133A microarray probe sets representing genes from the Angiogenesis (A), Inflammation (B), Integrin (C), and Wnt (D) “pathways” as annotated by PANTHER, across a set of 146 microarrays from our RCC experiment. For each “pathway”, up to four probe set clusters (red boxes) were selected and combined for subsequent re-clustering. (E) Another PANTHER “pathway” (Apoptosis) and one RCC-relevant “pathway” (HIF). Note the presence of less genes in these matrices compared to A-D and the absence of clear probe set clusters (except for cell lines in “Apoptosis”, indicated by the green bottom line), visually subdividing the matrix. [file 1471-2407-12-310-S2.pdf]

Additional file - Fig. S3

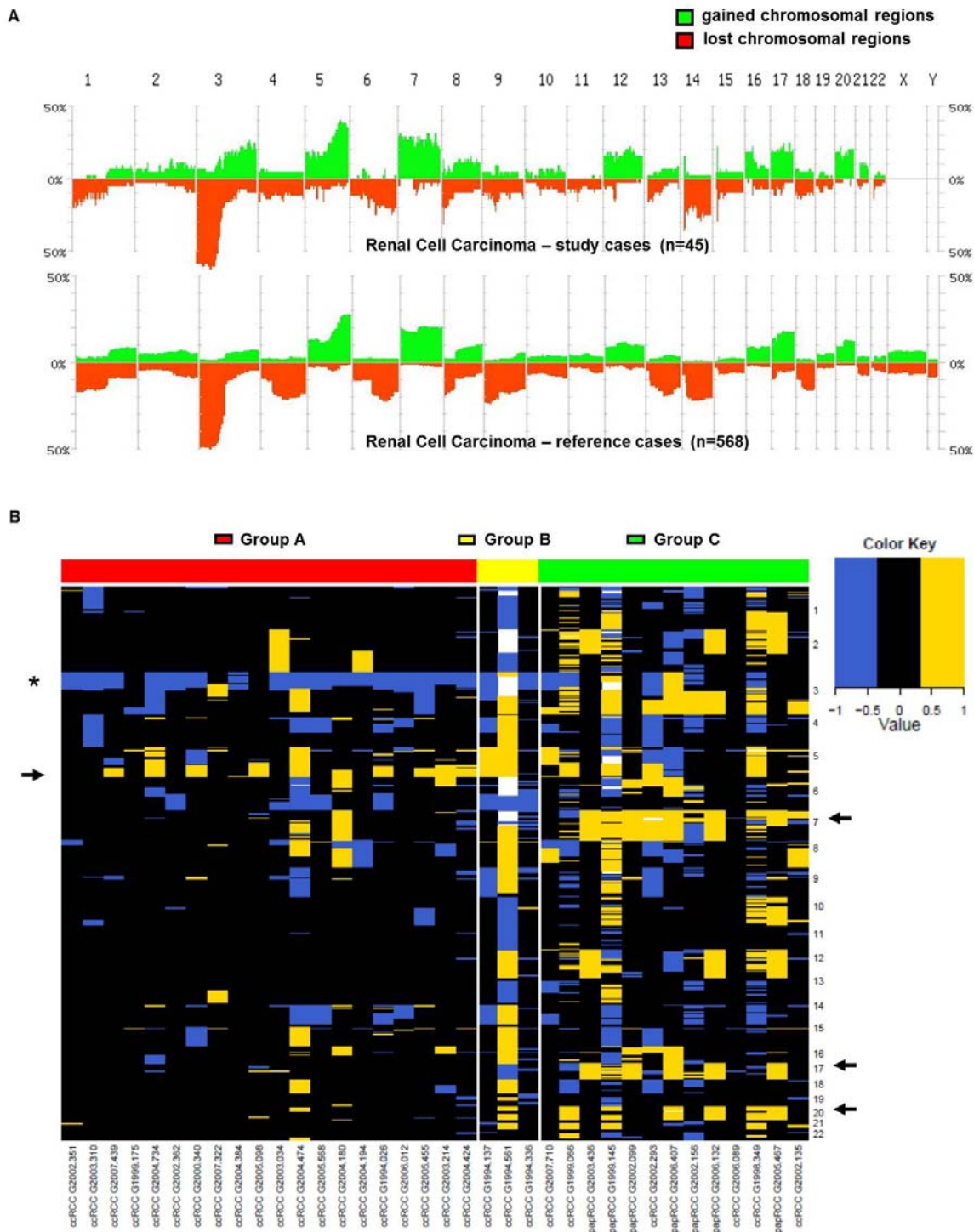

Supplement: Additional file 7 Figure S3 — The landscape of CNAs in RCC does not correlate with novel molecular subgroups. (A) Regional genomic CNAs in RCC shown as percentage of analyzed cases (genomic gains: yellow, up; losses: blue, down). Top: depiction of the overall CNAs in the 45 study cases; Down: published chromosomal and array CGH RCC data accessible through the Progenetix database (568 cases). Copy number variants (CNVs) were not filtered from the study case data besides application of a 100 kb size limit. Note the similar profiles. (B) Case specific regional copy number imbalances in 36 RCC study cases with regional genomic gain or loss status matched to 811 cytogenetic regions. The genomic profiles are randomly arranged within their subtypes. White areas indicate concurrent gain and loss in this cytoband. Note the appearance of known subtype-specific genomic alterations (3p deletions, 5q gains identifying clear cell RCC – asterisk and arrow/left side; gains of chromosomes 7, 17 and 20 identifying papillary RCC - arrows right side). [file 1471-2407-12-310-S7.pdf]

**Additional file - Fig. S4**

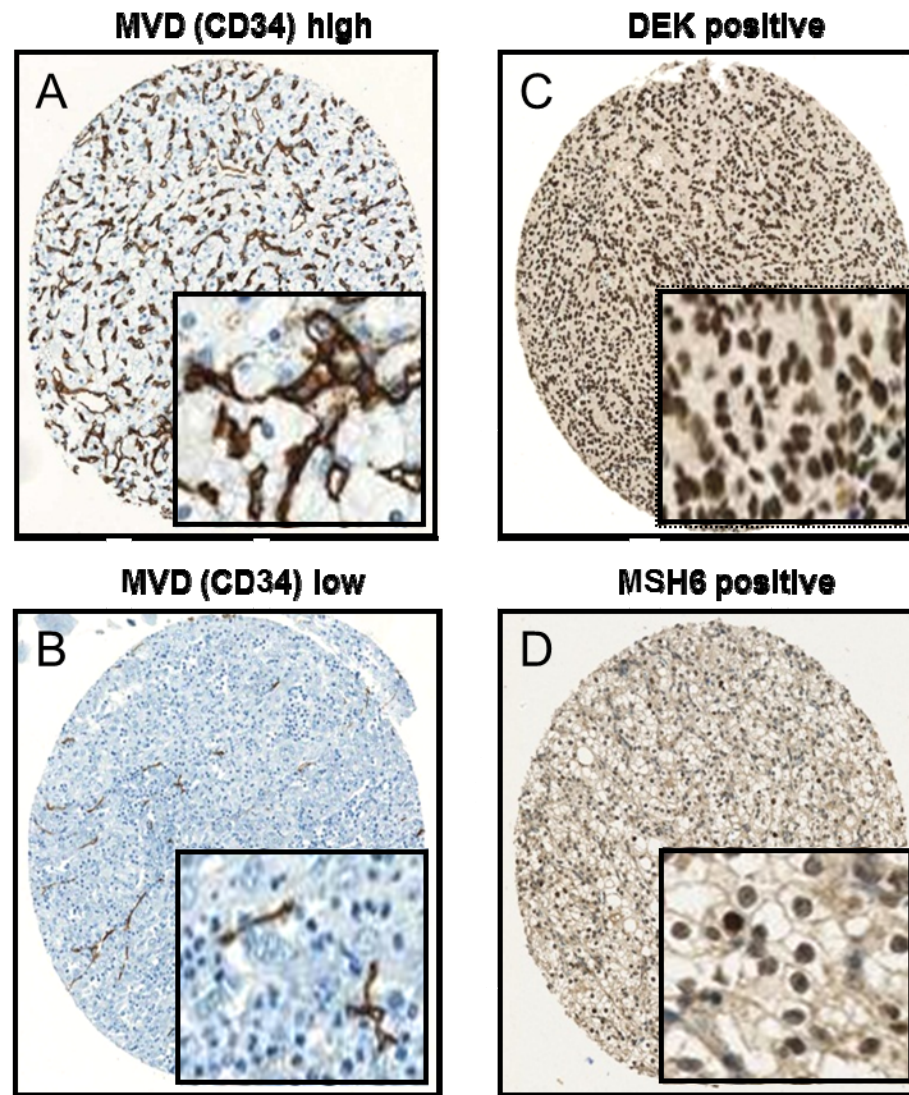

Supplement: Additional file 11 Figure S4 — Examples of immunostained RCC group-specific markers CD34, DEK and MSH6. ccRCC with CD34-stained vascular microvessels (A, B); ccRCC with strong nuclear DEK (C) and MSH6 (D) positivity. [file 1471-2407-12-310-S11.pdf]
