# Supplementary material for: Integrative genome-wide expression profiling identifies three distinct molecular subgroups of renal cell carcinoma with different patient outcome
Source: BMC Cancer. 2012 Jul 23;12:310. doi: 10.1186/1471-2407-12-310 (PMC3488567; doi:10.1186/1471-2407-12-310)

Additional file - Fig. S2

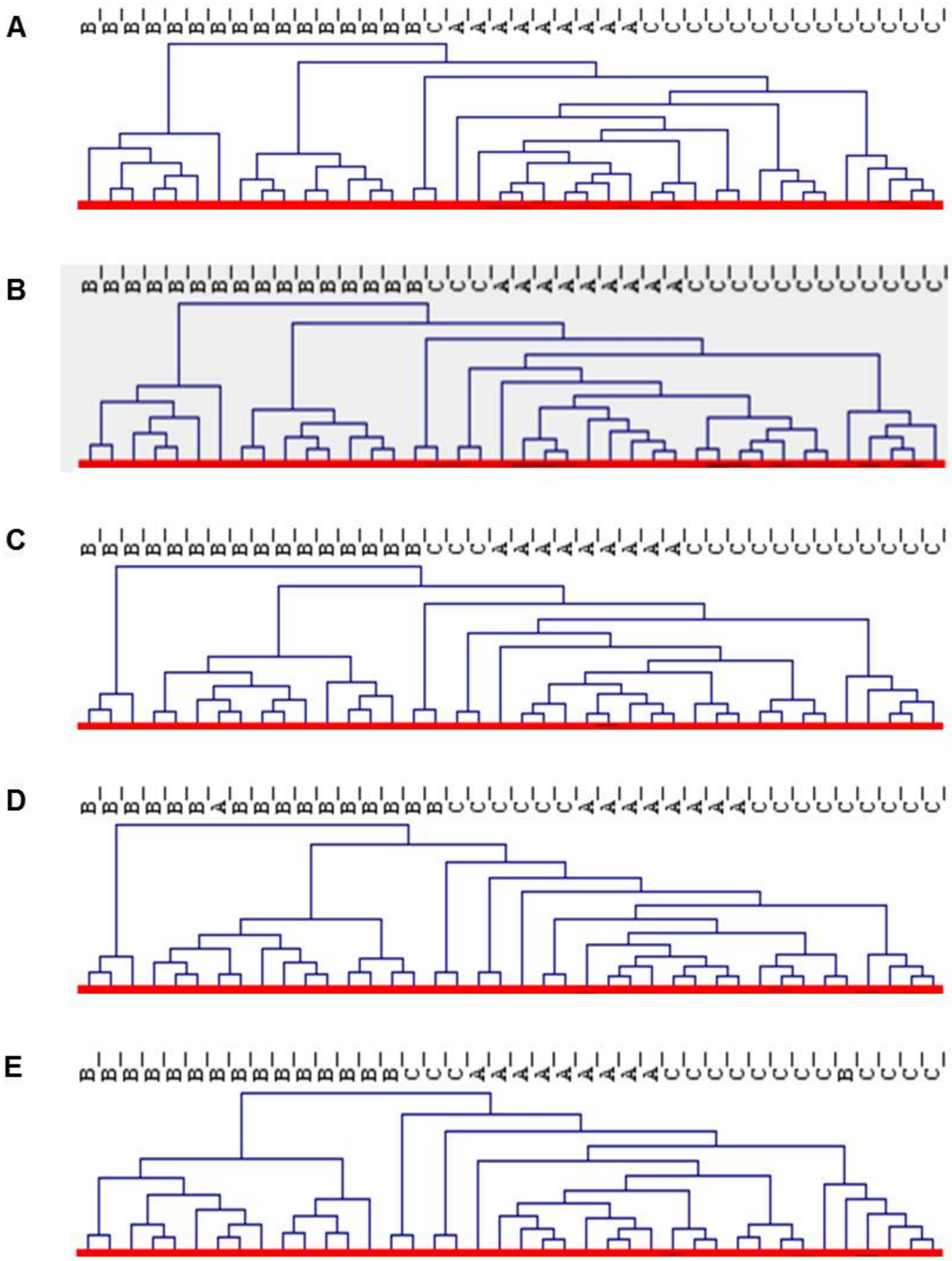

Supplement: Additional file 4 Figure S2 — The three RCC gene expression signatures spread genome-wide. Hierarchical clustering of 5 times arbitrarily chosen probe sets (each composed of ca. 660 genes) against group affiliated tumors (individual group-sample is labeled as A_, B_ or C_) (A-E). Note the tumor-group forming coincidence within the 5 independent analyses and the similarity with that shown in Figure 2A. [file 1471-2407-12-310-S4.pdf]
